# Near-complete genome sequences of five norovirus GII.4[P31] strains from coastal Kenya, 2019

**DOI:** 10.1128/mra.00748-26

**Published:** 2026-10-15

**Authors:** Arnold W. Lambisia, Clement S. Lewa, Parcelli Jepchirchir, Martin Mutunga, Charles N. Agoti

**Affiliations:** 1 Epidemiology and Demography Department, Kenya Medical Research Institute (KEMRI)–Wellcome Trust Research Programme (KWTRP) 118982, Nairobi, Kenya; 2 School of Health and Human Sciences, Pwani University 270495, Kilifi, Kenya; Katholieke Universiteit Leuven, Leuven, Belgium

**Keywords:** diarrhea, norovirus, amplicon-based, genomes, Kenya

## Abstract

There is limited norovirus genomic data from Africa. We report five near-complete norovirus genomes of genotype GII.4[P31], recovered from stool samples of children admitted with acute diarrhea in coastal Kenya during 2019. These expand the available genomic data resource for norovirus evolutionary studies, molecular assay improvement, and therapeutics development.

## ANNOUNCEMENT

Norovirus is a leading cause of viral acute gastroenteritis globally, disproportionately affecting young children (1, 2). Our previous work in coastal Kenya (2019–2022) detected norovirus GII in 10.2% of children admitted with acute diarrheal disease to Kilifi County Referral Hospital (KCRH) (3). Despite this significant disease burden, norovirus genomic data from Africa remain sparse, with only 51 near-complete genomes (>80% coverage) publicly available via GenBank as of June 2026.

Noroviruses are classified within the family *Caliciviridae* and the genus *Norovirus*. Their genome, ~7.5 kb in length, is a single-stranded positive-sense RNA molecule. Norovirus sequences fall into 10 genogroups (GI–GX), within which 49 genotypes have been identified to date (4). Of the 10 genogroups, GI, GII, and GIV are known to infect humans (4). Continuous expansion of the norovirus genomic data repository is essential in informing human vaccine development, improving both molecular diagnostics and partial or complete genome sequencing assays to enhance our understanding of its epidemiology, phylogeography, and phylodynamics, especially during its outbreaks.

Here, we report five near-complete norovirus GII.4[P31] genome sequences obtained from pediatric patients (<2 years old) admitted with diarrhea to KCRH, coastal Kenya, from February to August 2019 (Table 1). Briefly, total nucleic acid (TNA) was extracted from stool samples using QIAamp Fast DNA Stool Mini Kit (QIAGEN, UK) and screened via quantitative polymerase chain reaction using TaqMan Fast Virus 1-Step Master Mix (forward primer [5′-CARGARBCNATGTTYAGRTGGATGAG], reverse primer [5′-TCGACGCCATCTTCATTCACA], and probe [CY5-GAGGGSGATCGCRATCT-BHQ-2]) (3, 5). For whole-genome sequencing, the TNA for each analyzed sample was reverse-transcribed and amplified in three reaction tubes to generate 2.5–3.0 kb overlapping amplicons using SuperScript III One-Step RT-PCR System with Platinum Taq DNA Polymerase Kit (Invitrogen, UK) (primer sequences and PCR conditions described previously) (6). The resultant PCR products were pooled per sample in equal volumes, and sequencing libraries were prepared using the Illumina DNA-Flex Kit and sequenced on Illumina MiSeq (Illumina, USA), generating 75-bp paired-end reads. Sequencing adapters and low-quality reads (Phred score <30) were trimmed using QUASR v7.03 (7), and genomes were *de novo* assembled using SPAdes v3.13.2 (8) and annotated using Geneious Prime v1.0 (https://www.geneious.com). Default software parameters were used except where otherwise stated.

**TABLE 1:** Demographic and genomic characteristics of the five near-complete norovirus GII.4[P31] genome sequences from coastal Kenya^*a*^^,^^*c*^

Strain name	Sex	Age (months)	Collection date (day/mo/yr)	qPCR Ct value	Total reads generated	Genome length assembled	Average sequencing depth	Genome coverage (%)	GC content (%)	Closest match 1 (country: year, identity)	Closest match 2 (country: year, identity)	GenBank accession no.	SRA accession no.
KLF_NOV_001	M	9	22/02/2019	25.44	2,139,500	7,445	17,110	99.22	50	PV282524.1 (USA: 2015, 95.2%)	PV611446.1 (Malawi: 2016, 94.5%)	MW559994	SRR13626968
KLF_NOV_002	M	6	06/03/2019	22.19	1,624,198	7,443	6,580	99.12	50	PV282524.1 (USA: 2015, 95.2%)	PV611446.1 (Malawi: 2016, 94.5%)	MW559993	SRR13626967
KLF_NOV_003	F	12	09/03/2019	22.54	1,525,260	7,443	12,767	99.14	50.1	PV282524.1 (USA: 2015, 95.2%)	PV611446.1 (Malawi: 2016, 94.5%)	MW559991	SRR13626966
KLF_NOV_004	M	8	09/05/2019	18.30	2,230,880	7,443	17,969	99.16	50	PV282524.1 (USA: 2015, 95.2%)	PV611446.1 (Malawi: 2016, 94.5%)	MW559990	SRR13626965
KLF_NOV_005 (Hu/GII.4/P31/005/2019/KEN)^*b*^	F	14	07/08/2019	22.53	2,996,861	7,439	9,114	99.12	50.1	MT028542; (USA: 2018, 98.2%)	PV611445.1 (Malawi: 2015, 97.9%)	MW559992	SRR13626969

^*a*^ Abbreviations: bp, base pair; Ct, cycle threshold; qPCR, quantitative polymerase chain reaction; sex: M, male; F, female.

^*b*^ Strain KLF_NOV_005 represents the highly divergent GII.4[P31] lineage described as Hu/GII.4/P31/005/2019/KEN.

^*c*^ Note: Close references 1 and 2 exclude Kenya sequences to highlight the nearest non-local GenBank matches.

**Fig 1:**
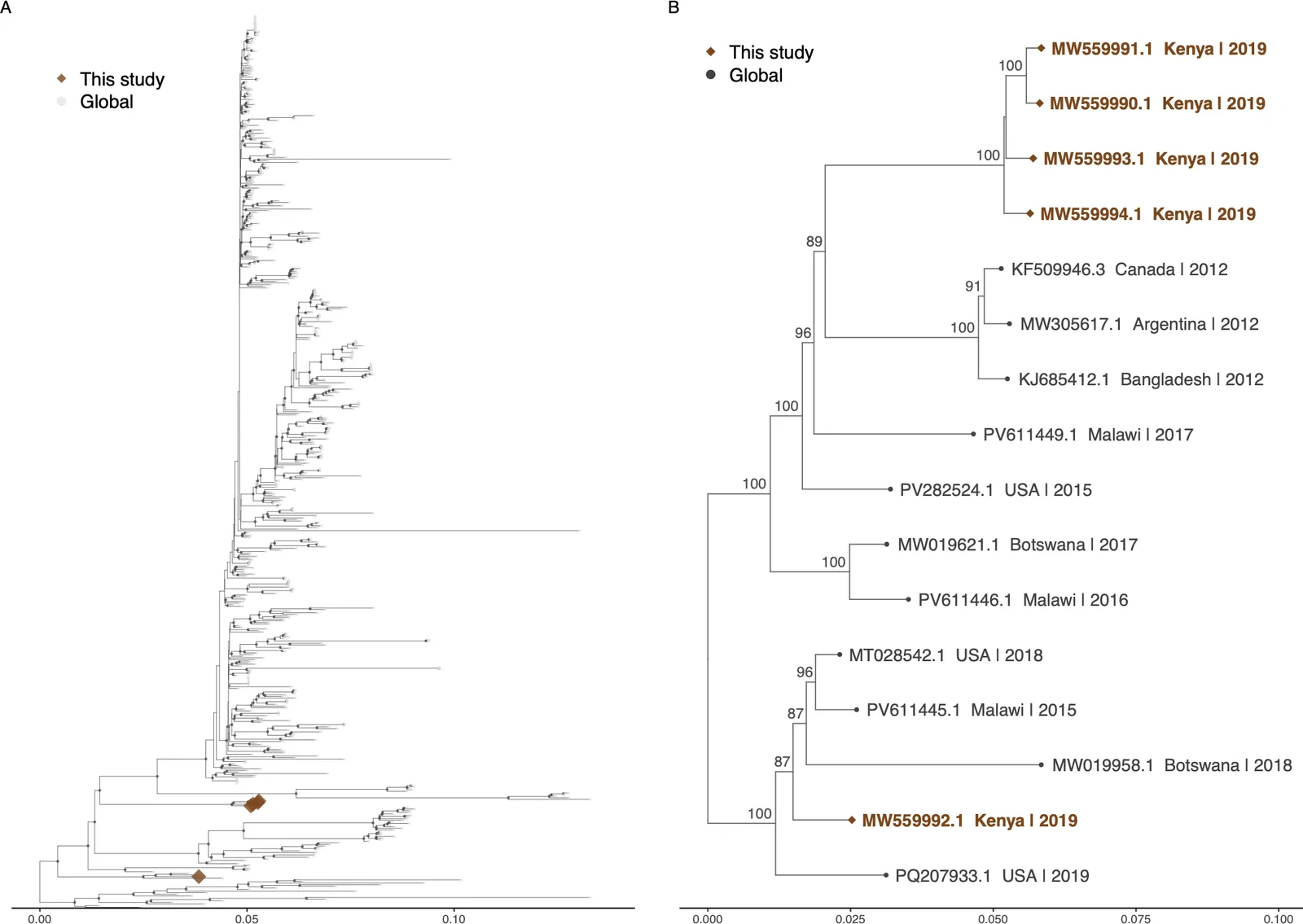
Maximum likelihood (ML) phylogenetic trees showing the phylogenetic placement of the five norovirus GII.4[P31] genomes from coastal Kenya within the global context. (**A**) An ML tree of 555 norovirus GII.4[P31] genomes (≥5,000 base pairs [bp]), including the five sequences in this study. All 4,400 norovirus GII sequences (≥5,500 bp) were retrieved from NCBI Virus and genotyped using RIVM Norovirus Genotyping Tool v2.0, and 555 genomes assigned as GII.4P[31] were retained. The 4,400-typing data and subsampling scripts can be found on GitHub (https://github.com/arnoldlambisia/Norovirus-GII-genotypes-summary) (9). Multiple sequence alignment was performed with MAFFT v7.526 (10). A maximum likelihood phylogeny was generated using IQ-TREE v3.1.1 (11) under the GTR + G + I + R5 model (selected as the best model by ModelFinder) and 1,000 ultrafast bootstrap replicates, and the tree was visualized and annotated using the ggtree v3.14 package in R v4.4.2 (12). Filled circles at internal nodes indicate a bootstrap support value of 100. (**B**) An ML tree showing Kenya sequences (brown tip points and labels) and their closest global sequences by pairwise nucleotide diversity. These were realigned, and a tree was generated as described above with the best model as GTR + F + G4. Bootstrap values are shown for nodes with ≥70 support. Global norovirus GII.4[P31] is shown in gray. The scale shows genetic distance in nucleotide substitutions per site. Re-organize columns as discussed.

The five assembled genomes achieved >99% genome coverage and were genotyped as GII.4[P31] using Norovirus Typing Tool v2.0 (https://mpf.rivm.nl/mpf/typingtool/norovirus) (Table 1). The four closely related sequences had 98.9%–99.5% (7,363–7,407 nucleotides) pairwise nucleotide similarity among each other, and one sample (Hu/GII.4/P31/005/2019/KEN) was divergent from the other four with 93.5%–93.6% (6,952–6,960 nucleotides) similarity. Phylogenetic analysis of the Kilifi sequences and contemporaneous global GII sequences revealed that the Kilifi sequences clustered closely with sequences from Canada, Argentina, and Bangladesh (2012) (Fig. 1). The divergent Kenyan sequence (Hu/GII.4/P31/005/2019/KEN) clustered with global GII.4[P31] sequences from the USA (2018–2019) with 97.9%–98.2% pairwise nucleotide similarity (Table 1 and Fig. 1). The pairwise nucleotide similarity and accession numbers of the top two closely related sequences in GenBank are provided in Table 1. These newly reported coastal Kenya sequences provide valuable data on norovirus GII diversity in an LMIC setting, supporting ongoing vaccine, diagnostic and sequencing assay development.

## Data Availability

The raw sequence data were deposited in the Sequence Read Archive (SRA) under BioProject accession number PRJNA699430 and SRA accession numbers SRR13626965–SRR13626969. The genome sequences generated here were deposited in GenBank under accession numbers MW559990–MW559994.
